# Headache in the adult population of Cameroon: prevalence estimates and demographic associations from a cross-sectional nationwide population-based study

**DOI:** 10.1186/s10194-024-01748-9

**Published:** 2024-03-21

**Authors:** Callixte Kuate Tegueu, Anastase Dzudie Tamdja, Franklin Kom, Blaise Forgwa Barche, Peter Ebasone, Mélanie Magnerou, Paul Mbonda, Jacques Doumbe, Andreas Husøy, Hallie Thomas, Timothy J. Steiner

**Affiliations:** 1 Department of Neurology, Douala Laquintinie Hospital, Douala, Cameroon; 2 Faculty of Medicine and Biomedical Sciences, University of Yaoundé, Yaoundé, Cameroon; 3 Department of Internal Medicine, Douala General Hospital, Douala, Cameroon; 4 Clinical Research Education, Networking and Consultancy (CRENC), Yaoundé, Cameroon; 5 Department of Neuromedicine and Movement Science, Norwegian University of Science and Technology (NTNU), Edvard Griegs Gate, Trondheim, Norway; 6 Department of Neurology, University of Copenhagen, Copenhagen, Denmark; 7 Division of Brain Sciences, Imperial College London, London, UK

**Keywords:** Epidemiology, Prevalence, Population-based study, Headache, Migraine, Tension-type headache, Medication-overuse headache, Cameroon, Sub-Saharan Africa, Global Campaign against Headache

## Abstract

**Background:**

Knowledge of headache prevalence, and the burdens attributable to headache disorders, remains incomplete in sub-Saharan Africa (SSA): reliable studies have been conducted only in Zambia (southern SSA) and Ethiopia (eastern SSA). As part of the Global Campaign against Headache, we investigated the prevalence of headache in Cameroon, in Central SSA.

**Methods:**

We used the same methodology as the studies in Zambia and Ethiopia, employing cluster-randomized sampling in four regions of Cameroon, selected to reflect the country’s geographic, ethnic and cultural diversities. We visited, unannounced, randomly selected households in each region, and randomly selected one adult member (aged 18–65 years) of each. Trained interviewers administered the Headache-Attributed Restriction, Disability and Impaired Participation (HARDSHIP) structured questionnaire, developed by an international expert consensus group and translated into Central African French. Demographic enquiry was followed by diagnostic questions based on ICHD-3 criteria.

**Results:**

Headache was a near-universal experience in Cameroon (lifetime prevalence: 94.8%). Observed 1-year prevalence of headache was 77.1%. Age- and gender-adjusted estimates were 76.4% (95% confidence interval: 74.9–77.9) for any headache, 17.9% (16.6–19.3) for migraine (definite + probable), 44.4% (42.6–46.2) for tension-type headache (TTH; also definite + probable), 6.5% (5.7–7.4) for probable medication-overuse headache (pMOH) and 6.6% (5.8–7.6) for other headache on ≥ 15 days/month (H15 +). One-day prevalence (“headache yesterday”) was 15.3%. Gender differentials were as expected (more migraine and pMOH among females, and rather more TTH among males). pMOH increased in prevalence until age 55 years, then declined somewhat. Migraine and TTH were both associated with urban dwelling, pMOH, in contrast, with rural dwelling.

**Conclusions:**

Headache disorders are prevalent in Cameroon. As in Zambia and Ethiopia, estimates for both migraine and TTH exceed global mean estimates. Attributable burden is yet to be reported, but these findings must lead to further research, and measures to develop and implement headache services in Cameroon, with appropriate management and preventative strategies.

## Background

Headache disorders should be taken seriously: they are leading causes of ill-health everywhere in the world. Migraine is recognized by the Global Burden of Disease (GBD) study as the second most disabling condition worldwide [1], although its estimated prevalence of 14–15% [2] is surpassed by the estimated 26% of tension-type headache (TTH) [3]. These are by far the most frequent of the primary headaches, but the disorders characterized by headache on ≥ 15 days/month (H15 +) are also common among the general population [3]. Imposing considerable disability, these include medication-overuse headache (MOH), which, although a secondary headache, develops as a complication of mismanaged migraine or TTH [4].

Action is required to mitigate the deleterious effects of these disorders on the health and wellbeing of both individuals and society. Promoting action, through raised awareness of need for action, has been a principal purpose of the Global Campaign against Headache [5], with better knowledge of the scale and scope of the burden of headache a prerequisite for this purpose. Accordingly, the Global Campaign, led by *Lifting The Burden* (LTB), a UK-registered non-governmental organization in official relations with the World Health Organization, has undertaken a series of national population-based studies [5]. Because most of the data contributing to GBD have come from high-income countries, these studies have focused on countries of low to middle income, and on regions outside Europe and North America.

The Republic of Cameroon in Central sub-Saharan Africa (SSA), with a population now of about 27 million, is a lower-middle-income country [6, 7]. Despite economic growth in some regions, poverty is on the rise [6]. This is most prevalent in rural areas, which are especially affected by a shortage of jobs, poor school and health-care infrastructures, and a lack of clean water and sanitation [6, 7]. While Cameroon is therefore a country of great inequality [6], health care is extremely limited for the majority of the population [8].

Two LTB-led studies have been conducted in SSA: one in Zambia, in southern SSA [9], and one in Ethiopia, in eastern SSA [10]. Both have shown highly prevalent and burdensome headache [9, 10]. In Cameroon, knowledge of headache is almost entirely lacking, with none deriving from population-based studies according to a search of the PubMed database. It is well recognized that headache is a ubiquitous presenting symptom of diseases such as malaria and HIV-AIDS, which affect large numbers of people in Cameroon, but these are secondary headaches. Their burdens are attributable to the underlying disorders, and they are not the focus of this study. Globally, migraine, TTH and MOH far outweigh the secondary headaches as contributors to population ill health [11]. Our own evidence, from a survey conducted 15 years ago within the neurology outpatient department of the Yaoundé Central Hospital, is that primary headaches are first among presenting complaints: one third (33.5%) of all patients seen in 12 months [unpublished]. In the big cities of Cameroon, such as Yaoundé, headache disorders are managed in specialized services such as neurology. While this survey provides evidence that headache disorders are a significant call upon health resources in Cameroon, it tells nothing of what is happening in the population, and offers no picture of headache countrywide. There are in Cameroon no data on referral paths to the few existing neurology services (including who is referred for the management of headache, when and why), and none on the profile of self-referring patients. There are no data on who can afford care, in a country where poor access to health care [8] leads to reliance on traditional medicines, and poverty teaches the harsh lesson that disease and pain are to be tolerated.

The aim of this study was to fill this knowledge gap. From a national population-based survey, we generated prevalence estimates for migraine, TTH, probable MOH (pMOH) and other H15 + , the headache disorders with public-health importance. In addition, we investigated the associations of each headache type with basic demographics.

## Methods

### Ethics

Approval was obtained from the Cameroon National Ethics Committee (reference n^0^ 2019/04/4458/CE/CNERSH/SP) before commencement of the study. All participants were informed of the nature and purpose of the study and gave oral consent prior to enrolment. The study was conducted in accordance with the Declaration of Helsinki [12].

All interviews were conducted in private, and responses kept confidential. All data were held and managed in accordance with data-protection legislation.

### Study design

This was a nationwide cross-sectional study of the adult population of Cameroon using established methodology [13]. It was conceived by LTB, and implemented locally by Clinical Research Education, Networking and Consultancy (CRENC), a well-established clinical research organization in Cameroon.

Nationals of Cameroon aged 18–65 years were eligible, and randomly selected for inclusion through a process of cluster-sampling. Trained interviewers followed a structured questionnaire.

The design incorporated a pre-pilot study to test acceptability of the questionnaire to potential participants, and a pilot study to ensure the methods would work.

#### Pre-pilot study

This study was clinic-based, conducted in Douala over the course of one month, with 40 participants aged 18–65 years in an approximately equal mix of patients presenting with headache and accompanying persons not complaining of headache. It used a draft adaptation of the questionnaire in the original English language, translated, when necessary, at point of application by the interviewers (physicians with full understanding of its meaning and purpose). Questions were reworded or restructured where necessary, and the questionnaire finalized prior to translation.

#### Questionnaire

We used the Headache-Attributed Restriction, Disability, Social Handicap and Impaired Participation (HARDSHIP) questionnaire developed by LTB [14], with demographic questions followed by headache screening questions (“have you ever had headache?” and “have you had headache during the last year?”). Diagnostic questions based on ICHD-3 [15] were asked of all those responding “yes” to the latter, with focus on the most bothersome headache when more than one type was reported. Additional questions asked about headache yesterday (HY): “did you have a headache yesterday?” and “was this the same type of headache as the one you have just been describing?”, which enabled estimation of point prevalence [13]. Further questions were on headache-attributed burden, but these are not reported here.

In accordance with LTB’s translation protocol for lay documents [16], the questionnaire was translated into Central African French, the principal language of Cameroon, before use in the pilot study.

#### Study areas

The population of Cameroon are, essentially, from four clans, introducing a wide cultural mix: the Sawas (from the Littoral, and Southwest), the grass-landers (from the West, and Northwest), the Fulani’s (from Adamawa, North and Far North) and the Bantus (from the Centre, South and East regions). We conducted the survey in both urban and rural areas of four of the ten regions of the country, fairly reflecting its ethnic and cultural diversities:

- Centre region: Yaoundé (urban); Mfou and Ngoumou (rural: 60 and 57 km from Yaoundé respectively);
- Littoral region: Douala (urban); Nkongsamba (rural areas 143 km from the town of Douala);
- West region: Bafoussam (urban); Bandjoun, Bayangam, Baham and Bangou (rural areas 16-34 km distant from the town of Bafoussam);
- Adamawa region: Ngaoundere (urban); Ngaoundal (rural areas 182 km from the town of Ngaoundere).

These four regions were home to almost half of the country’s population (Centre 18.2%, Littoral 14.8%, West 9.2% and Adamawa 5.2%) in the most recent (2005) national census [17].

#### Interviewers

The 15 interviewers were physicians, senior medical students, senior university students in biology, or nurses. All attended a two-day training session at CRENC’s office in Douala, including epidemiological and clinical aspects of headache disorders, and theoretical and practical aspects of the study design and purpose. Training included supervised interviews to ensure competence.

These interviewers conducted the pilot study, then continued with the main study.

#### Pilot study

The pilot study, completed during April 2019, tested the translated questionnaire in the field for ease of use, comprehensibility and lack of ambiguity. It also provided estimates of the non-participating proportion.

Communities were convenience-selected in each of the four study areas. A total of 160 biologically unrelated adults (aged 18–65 years), 40 from each area, and 80 urban- and 80 rural-dwellers overall, were surveyed using a mixture of convenience and purposive sampling.

### Main study

This was completed during June to August 2019.

#### Sampling

The overall population distribution of Cameroon in 2005 (in the most recent census [17]) was 51.2% rural, 48.8% urban, but urbanization was much higher in Littoral (92.6%) and Centre regions (71.9%). To maintain sufficient numbers for urban/rural comparisons, the rural populations in Littoral and Centre regions were oversampled (40%).

The sampling procedure was designed to accommodate multiple families living in single dwelling-places in unplanned settlements. We anticipated access obstacles in the cities from uncontrolled and poor housing, while rural areas, though less challenging from these perspectives, required more energy to access. The steps in either case were: (1) selection of health areas within each region, reflecting the region’s diversity; (2) a census, and listing, of dwellings in the selected health areas; (3) random selection of one or more blocks or circumscribed areas of dwellings and, within these blocks or areas, random selection of dwellings; (4) cold-calling at selected dwellings; (5) count of biologically-unrelated families living in each; (6) listing of all individuals in each family, representing a household (defined as a group of people eating from the same pot); (7) random selection by the lottery method of one eligible participant from each household. This person only was included in the sample. If unwilling or unable to participate, he or she was regarded as a non-participant, and not replaced from that household.

When a selected participant was not present, an appointment was made for the interviewer to return. Where the door to a selected dwelling was not answered at first visit, two further attempts were made before the dwelling was excluded and replaced by another according to the sampling algorithm.

The survey continued until a total in excess of 3,000 participants were successfully interviewed. Methodological guidelines recommend a minimum sample size of *N* = 2,000 [13].

#### Quality control

Interviewers checked the responses for completeness before ending each visit. They were supervised in each of the four regions by one of the physician investigators, who made random unannounced inspections, in the field, of the interviewers’ work-quality.

The principal investigator checked completed questionnaires as the study proceeded.

#### Data management

Before data entry, questionnaires were scrutinised for inconsistencies, wrong entries, illegible markings and missed entries. All data were then entered twice, independently, by two groups of 7th-year medical students under supervision by two epidemiologists, into a secure database provided by CRENC using Epi Info version 7.2. The two datasets were compared using Microsoft Excel version 16 as a secondary platform, and any discrepancies resolved by cross-checking against the original questionnaires.

Paper records were stored securely for quality checks and data confirmation.

### Analysis

Gender was recorded as either male or female. Age was reported as a continuous variable, but categorized for association analyses (18–25, 26–35, 36–45, 46–55 and 56–65 years). Habitation was recorded as either urban or rural. Marital status was recorded as single, married, separated, divorced or widowed, and the last three combined for analyses. Education level was recorded as none, primary school, secondary school or college/university. Annual household income was recorded in Central African francs (XAF) in three categories (< 50,000; 50,001–100,000; > 100,000) (in June 2019, USD 1 = XAF 583).

#### Headache diagnoses

Diagnoses were not made by the interviewers but later derived algorithmically [14]. Individuals reporting H15 + were first identified, with those reporting consumption of acute medication on ≥ 15 days/month (assumed for the vast majority to be restricted to simple analgesics) considered to have pMOH and the remainder diagnosed as “other H15 + ”. To all others, the ICHD-based criteria were applied in the following order: definite migraine, definite TTH, probable migraine, probable TTH [15]. Definite and probable migraine were combined in analyses, as were definite and probable TTH.

#### Statistics

Demographic data were analyzed descriptively, with continuous variables summarized as means and standard deviations (SDs) and categorical variables as frequencies and percentages with confidence intervals (CIs) where appropriate. The male–female and urban–rural ratios within the sample were compared with those of the general population of Cameroon aged 18–65 years using chi-squared tests.

Prevalences were estimated as percentages (%) with 95% CIs. Observed 1-year prevalences of all headache and of each headache type were adjusted for age and gender. Observed point prevalence of all headache was compared with predicted point prevalence calculated from observed 1-year prevalence and reported mean headache frequency (days/month).

Associations between each headache type (dependent variable) and demographic and social status variables (independent variables) were investigated using bivariate and multivariate analyses (the latter including all independent variables), calculating odds ratios (ORs) and adjusted ORs (aORs) with 95% CIs.

We considered *p* < 0.05 to be significant.

IBM-Statistical Package for Social Sciences statistical software (SPSS) version 28 (SPSS Inc., Chicago, IL) was used for all analyses except the adjusted prevalences, for which Microsoft Excel version 16 was used.

## Results

### Description of sample

We included 3,100 individuals from the four regions as follows: Adamawa 27.1%, Centre 24.3%, Littoral 24.5% and West 24.2%. Females were somewhat overrepresented (54.6%) compared to the gender distribution in the country (50.4%; chi-squared = 21.8, *p* < 0.001), but mean age (34.9 years; males 35.9; females 34.0) was close to that of the population aged 18–65 years (34.2 years). Habitation (57.5% urban) perfectly matched that of Cameroon’s population (57% urban).

Non-participating proportion in the pilot study was 16.7%, with some regional variation (Centre 12.8%, Littoral 17.5%, West 21.7% [notably, 3.3% in rural areas, 40.0% in urban], Adamawa 15.0%).

### Prevalence

In total, 94.8% of participants reported ever having (any) headache, with no difference between males (94.2%; 95% CI: 92.8–95.3) and females (95.3% [94.2–96.3]). One-year prevalence of any headache (77.1%), on the other hand, was higher among females (79.9% [77.9–81.8]) than males (73.6% [71.2–75.9]).

Table 1 shows the observed 1-year prevalence of all headache and of each headache type, overall and by gender. Only 1.0% of reported headaches were unclassified.

**Table 1:** Observed 1-year prevalence of headache types by gender

**Headache type**	**Overall** % [95% CI]	**Male** % [95% CI]	**Female** % [95% CI]
All headache	77.1 [75.5–78.5]	73.6 [71.2–75.9]	79.9 [77.9–81.8]
Migraine	18.9 [17.5–20.3]	15.9 [14.1–17.9]	21.4 [19.5–23.4]
Definite	5.6 [4.9–6.5]	4.3 [3.3–5.5]	6.8 [5.7–8.1]
Probable	13.3 [12.1–14.5]	11.7 [10.0–13.5]	14.6 [13.0–16.4]
TTH	44.0 [42.2–45.8]	47.8 [45.1–50.4]	40.9 [38.5–43.3]
Definite	36.3 [34.6–38.0]	40.2 [37.7–42.8]	33.0 [30.7–35.3]
Probable	7.7 [6.8–8.7]	7.5 [6.2–9.0]	7.9 [6.7–9.3]
pMOH	6.5 [5.6–7.4]	4.0 [3.0–5.2]	8.5 [7.2–9.9]
Other H15 +	6.7 [5.9–7.7]	5.0 [4.0–6.4]	8.1 [6.9–9.5]

*TTH* tension-type headache, *pMOH* probable medication-overuse headache, *H15* + headache on ≥ 15 days/month

TTH was the most commonly reported headache type (by 44.0% of participants), with migraine second (18.9%) (Table 1). H15 + was reported by 12.2% of participants, of whom 6.5% were classified as pMOH. Since the sample was well matched to the population, age- and gender-adjusted 1-year prevalences were similar to the observed: any headache 76.4% (74.9–77.9), TTH 44.4% (42.6–46.2), migraine 17.9% (16.6–19.3), pMOH 6.5% (5.7–7.4), other H15 + 6.6% (5.8–7.6).

Headache yesterday (HY) was reported by 15.3% (14.1–16.6) of the total sample, 19.8% of those with headache last year. HY was more common among those with migraine (21.8%) than those with TTH (10.5%), and, as expected, much more common among those with pMOH (45.5%) or other H15 + (37.5%). Based on the observed prevalence of any headache (77.1%) and the reported mean headache frequency (6.7 days/month), the predicted point prevalence of any headache was 17.2%.

### Associations

Female preponderance was noted in bivariate analyses for all headache types except TTH, and confirmed in multivariate analyses, although it was not significant for other H15 + : migraine 21.4% *vs* 15.9% (aOR = 1.6; *p* < 0.001); pMOH 8.5% *vs* 4.0% (aOR = 1.9; *p* < 0.001); other H15 + 8.1% *vs* 5.0% (aOR = 1.3; *p* = 0.08). TTH was more prevalent among males (48.2%) than females (41.1%), but this just missed significance in the adjusted (multivariate) analysis model (aOR = 0.9; *p* = 0.06). Tables 2 and 3 show these and other associations with demographic and social status variables, in bivariate and multivariate analyses.

**Table 2:** Bivariate analyses of associations between headache types and demographic variables

Variable	Migraine	TTH	pMOH	Other H15 +
	Odds ratios [95% CIs]			
**Gender**				
Male (*n* = 1,407)	reference	reference	reference	reference
Female (*n* = 1,693)	**1.4 [1.2–1.7] *p* < 0.001**	**0.8 [0.7–0.9] *p* < 0.001**	**2.2 [1.6–3.1] *p* < 0.001**	**1.7 [1.2–2.2] *p* < 0.001**
**Age** (years)				
18–25 (*n* = 904)	reference	reference	reference	reference
26–35 (*n* = 875)	1.2 [1.0–1.6] *p* = 0.09	1.0 [0.8–1.2] *p* = 0.96	1.3 [0.9–2.0] *p* = 0.17	**0.6 [0.4–0.8] *p* = 0.003**
36–45 (*n* = 655)	**1.3 [1.0–1.7] *p* = 0.04**	0.9 [0.8–1.1] *p* = 0.49	1.3 [0.8–2.0] *p* = 0.22	**0.6 [0.4–0.9] *p* = 0.02**
46–55 (*n* = 413)	1.0 [0.7–1.4] *p* = 0.85	0.9 [0.7–1.1] *p* = 0.40	**1.9 [1.2–3.0] *p* = 0.004**	0.6 [0.4–1.0] *p* = 0.06
56–65 (*n* = 252)	0.9 [0.6–1.3] *p* = 0.41	1.2 [0.9–1.6] *p* = 0.21	1.5 [0.8–2.6] *p* = 0.18	**0.3 [0.2–0.7] *p* = 0.002**
**Habitation**				
Urban (*n* = 1,784)	reference	reference	reference	reference
Rural (*n* = 1,316)	**0.7 [0.6–0.9] *p* < 0.001**	**0.6 [0.5–0.7] *p* < 0.001**	**2.5 [1.9–3.4] *p* < 0.001**	**1.3 [1.0–1.8] *p* = 0.04**
**Marital** status^a^				
Single (*n* = 1,295)	reference	reference	reference	reference
Married (*n* = 1,584)	**1.4 [1.2–1.7] *p* < 0.001**	0.9 [0.8–1.0] *p* = 0.08	**0.7 [0.5–0.9] *p* = 0.02**	0**.7 [0.5–0.9] *p* = 0.01**
Widowed, separated or divorced (*n* = 212)	1.0 [0.7–1.5] *p* = 0.93	0.8 [0.6–1.1] *p* = 0.13	**2.1 [1.3–3.2] *p* = 0.001**	0.9 [0.5–1.7] *p* = 0.87
**Education level**^**b**^				
None (*n* = 169)	**1.7 [1.1–2.6] *p* = 0.009**	**0.4 [0.2–0.5] *p* < 0.001**	**2.2 [1.3–3.9] *p* = 0.005**	0.9 [0.5–1.7] *p* = 0.73
Primary (*n* = 489)	1.4 [1.0–1.8] *p* = 0.07	**0.5 [0.4–0.6] *p* = < 0.001**	1.3 [0.8–2.1] *p* = 0.24	0.8 [0.6–1.4] *p* = 0.54
Secondary (*n* = 1,706)	**1.6 [1.2–2.0] *p* < 0.001**	**0.8 [0.6–0.9] *p* = 0.002**	1.1 [0.7–1.5] *p* = 0.74	0.8 [0.6–1.1] *p* = 0.10
University/college (n = 721)	reference	reference	reference	reference
**Household income** (XAF)^c^				
< 50,000 (*n* = 1,758)	**0.7 [0.6–0.9] *p* = 0.01**	**0.7 [0.6–0.9] *p* = 0.001**	**1.8 [1.2–2.8] *p* = 0.008**	**2.4 [1.5–3.8] *p* < 0.001**
50,001–100,000 (*n* = 619)	1.0 [0.8–1.3] *p* = 0.98	0.9 [0.7–1.1] *p* = 0.36	1.3 [0.8–2.2] *p* = 0.36	1.0 [0.5–1.8] *p* = 0.99
> 100,000 (*n* = 608)	reference	reference	reference	reference

*TTH* tension-type headache, *pMOH* probable medication-overuse headache, *H15* + headache on ≥ 15 days/month

^a^9 missing

^b^15 missing

^c^124 missing; significant values are emboldened

**Table 3:** Multivariate analyses of associations between headache types and demographic variables

Variable	Migraine	TTH	pMOH	Other H15 +
	Adjusted odds ratios^a^ [95% CIs]			
**Gender**				
Male	reference	reference	reference	reference
Female	**1.6 [1.3–2.0] *p* < 0.001**	0.9 [0.7–1.0] *p* = 0.06	**1.9 [1.4–2.8] *p* < 0.001**	1.3 [1.0–1.8] *p* = 0.08
**Age** (years)				
18–25	reference	reference	reference	reference
26–35	1.0 [0.8–1.3] *p* = 0.96	1.0 [0.8–1.3] *p* = 0.86	**1.7 [1.1–2.6] *p* = 0.02**	0.7 [0.5–1.1] *p* = 0.15
36–45	1.0 [0.7–1.4] *p* = 0.96	1.0 [0.8–1.3] *p* = 0.75	**2.0 [1.2–3.3] *p* = 0.009**	0.8 [0.5–1.3] *p* = 0.42
46–55	0.8 [0.6–1.2] *p* = 0.26	1.1 [0.8–1.5] *p* = 0.44	**2.6 [1.5–4.5] *p* = 0.001**	0.8 [0.5–1.5] *p* = 0.56
56–65	0.7 [0.6–1.2] *p* = 0.10	**1.5 [1.1–2.2] *p* = 0.02**	1.8 [0.9–3.8] *p* = 0.10	**0.3 [0.1–0.9] *p* = 0.03**
**Habitation**				
Urban	reference	reference	reference	reference
Rural	**0.7 [0.4–0.9] *p* = 0.003**	**0.7 [0.6–0.8] *p* < 0.001**	**2.0 [1.5–2.8] *p* < 0.001**	1.1 [0.8–1.5] *p* = 0.52
**Marital status**				
Single	reference	reference	reference	reference
Married	1.2 [1.0–1.6] *p* = 0.07	0.8 [0.7–1.0] *p* = 0.09	**0.6 [0.4–0.8] *p* = 0.003**	1.1 [0.7–1.6] *p* = 0.64
Widowed, separated or divorced	1.0 [0.6–1.6] *p* = 0.97	0.9 [0.6–1.3] *p* = 0.53	1.1 [0.6–2.0] *p* = 0.74	1.3 [0.6–2.7] *p* = 0.52
**Education level**				
None	**1.9 [1.2–3.0] *p* = 0.006**	**0.4 [0.3–0.6] *p* < 0.001**	1.7 [0.9–3.2] *p* = 0.13	0.7 [0.3–1.4] *p* = 0.26
Primary	**1.6 [1.1–2.2] *p* = 0.01**	**0.6 [0.4–0.8] *p* < 0.001**	0.9 [0.5–1.5] *p* = 0.62	0.6 [0.4–1.0] *p* = 0.07
Secondary	**1.6 [1.3–2.1] *p* < 0.001**	**0.8 [0.7–1.0] *p* = 0.02**	0.9 [0.6–1.4] *p* = 0.80	**0.6 [0.4–0.9] *p* = 0.02**
University/college	reference	reference	reference	reference
**Household income** (XAF)				
< 50,000	**0.7 [0.5–0.9] *p* = 0.004**	1.0 [0.8–1.2] *p* = 0.76	1.2 [0.7–2.0] *p* = 0.46	**2.2 [1.3–3.7] *p* = 0.003**
50,001–100,000	0.9 [0.7–1.2] *p* = 0.46	1.0 [0.8–1.3] *p* = 0.81	1.0 [0.6–1.8] *p* = 0.90	1.1 [0.6–2.0] *p* = 0.85
> 100,000	reference	reference	reference	reference

*TTH* tension-type headache, *pMOH* probable medication-overuse headache, *H15* + headache on ≥ 15 days/month; significant values are emboldened

^a^Adjusted for all other variables

Migraine was most prevalent among those aged 36–45 years, an association that was significant in bivariate (OR = 1.3; *p* = 0.04) (Table 2) but not multivariate analysis (Table 3). TTH varied with age only in multivariate analysis (most prevalent among those aged 56–65 years: aOR = 1.5; *p* = 0.02) (Table 3). pMOH peaked in prevalence among those 46–55 years (OR = 1.9; *p* = 0.004) (Table 2), more robustly in multivariate analysis (aOR = 2.6; *p* = 0.001) (Table 3). Other H15 + was least prevalent among those aged 56–65 years (aOR = 0.3; *p* = 0.03) (Table 3).

Both migraine (OR = 0.7; *p* < 0.001) and TTH (OR = 0.6; *p* < 0.001) were less prevalent in rural areas, contrary to pMOH (OR = 2.5; *p* < 0.001) and other H15 + (OR = 1.3; *p* = 0.04) (Table 2). All these associations except the last survived adjustment in multivariate analyses (migraine: aOR = 0.7; *p* = 0.003; TTH: aOR = 0.7; *p* < 0.001; pMOH: aOR = 2.0; *p* < 0.001) (Table 3).

Married participants tended to have more migraine but less pMOH and other H15 + than single, but only the negative association with pMOH survived adjustment (aOR = 0.6; *p* = 0.003) (Tables 2 and 3).

Participants with university or college education were least likely to have migraine but most likely to have TTH; prevalence of TTH was positively associated with educational level across its spectrum (Tables 2 and 3). Those with no education had most migraine (aOR = 1.9; *p* = 0.006), least TTH (aOR = 0.4; *p* < 0.001) and, on bivariate analysis, most pMOH (OR = 2.2; *p* = 0.005) (Tables 2 and 3).

In bivariate analyses, migraine and TTH were positively associated, and pMOH and other H15 + negatively, with household income. Thus, those with the lowest income had least migraine (OR = 0.7; *p* = 0.01) and TTH (OR = 0.7; *p* = 0.001) but most pMOH (OR = 1.8, *p* = 0.008) and other H15 + (OR = 2.4; *p* < 0.001). After adjustment, only the findings for migraine (aOR = 0.7; *p* = 0.004) and other H15 + (aOR = 2.2; *p* = 0.003) remained significant.

## Discussion

This adult population-based study in Cameroon, the first of its type in Central SSA, found that headache was a near-universal experience (lifetime prevalence: 94.8%). As expected, the 1-year prevalence of headache was lower, but still very high (observed: 77.1%; age and gender-adjusted: 76.4%). Malaria, endemic in Cameroon, may largely have accounted for the difference between lifetime and 1-year prevalences, but we did not investigate this. TTH was the most common headache type (age and gender-adjusted 1-year prevalence 44.4%), followed by migraine (17.9%), but, notably, both pMOH (6.5%) and other H15 + (6.6%) were also prevalent. One in seven (15.3%) of our sample, had headache on the day prior to the interview (HY); a similar proportion, presumably, have headache on any day.

The prevalence of pMOH and other H15 + combined (*ie*, all headache with a frequency of ≥ 15 days/month) was very high (13.1% overall, 16.6% and 9.0% among females and males respectively), surpassing the 11.5% seen in Zambia in a similar study conducted by LTB [9]. Prevalences of pMOH were similar in the two countries (Cameroon 6.5%, Zambia 7.1% [9]). In marked contrast to Zambia, where pMOH was much more of an urban problem than rural (OR = 8.6) [9], in Cameroon pMOH was associated with rural dwelling (OR = 2.5). In Zambia, urban association was explained by poor access to health care and lack of health education everywhere, but much easier access in towns to over-the-counter (OTC) medications [9]. Both Cameroon and Zambia are lower-middle-income countries, with similar health-care deficiencies and inequalities. Zambia, however, was considerably less urbanized (40% at the time of its survey [9]) than Cameroon (57%), creating a greater barrier to access to OTC medications. There was also some potential for confounding. In the bivariate analyses, pMOH in Cameroon was highly associated not only with rural dwelling but also with no education and low household income. All three of these are highly associated with each other, and, in the multivariate analyses, only rural dwelling remained significant, as noted. Furthermore, a majority of those living in rural areas in Cameroon were female [18]. Female gender was itself associated with pMOH (aOR = 1.9), while females would tend also to be less well educated.

Two methodological considerations might, in addition, have been relevant to these estimates. In both countries, it was understood that very few people had access to other than simple analgesics, and a conservative threshold for medication overuse was applied (≥ 15 days/month in Cameroon and > 3 days/week in Zambia [9] rather than ≥ 10 days/month [15]). The estimated proportions with pMOH would almost certainly have been higher with the lower threshold, but erroneously so. On the other hand, since migraine is the main progenitor of pMOH, and our diagnostic algorithm prioritized the latter while allowing only a single diagnosis per respondent, there was potential for interaction. In other words, many of those diagnosed with pMOH might otherwise have been diagnosed with migraine (*ie*, migraine prevalences were probably somewhat underestimated).

This survey recorded a rather higher proportion of other H15 + in Cameroon (6.6%) than was reported in Zambia (4.4% [9]). A third and probably important methodological consideration here is that in Zambia, one of the earliest of LTB’s studies, the screening question was “In the last year, have you had headache that was not part of another illness?” Later studies recognized the greater value of a neutral question [13] (“have you had headache during the last year?” was used in Cameroon). One-year prevalence of any headache was 76.4% in Cameroon, but only 61.6% was reported in Zambia in response to its restrictive screening question [9]. In Zambia, headache attributed to malaria (also endemic there) was likely to have been excluded, but that was not so in Cameroon, where, if present, it would probably have been categorized as other H15 + (reported by 6.6%).

Other demographic associations in Cameroon offered no new insights. Gender differentials were as expected (more migraine and pMOH among females, and rather more TTH among males). With regard to age, the expected increase in migraine prevalence, then decline (after age 45 years), was apparent only in bivariate analysis. pMOH increased in prevalence until age 55 years, then declined somewhat. Interaction between migraine and pMOH diagnoses (referred to above) might have been a factor in these analyses.

In contrast to pMOH, migraine and TTH were both associated with urban dwelling. This was notwithstanding a strong negative association between migraine and educational level, the latter positively associated with urban dwelling. (TTH, on the other hand, was negatively associated with educational level.) It is easy to speculate that rural dwelling is more peaceful and less stressful, but again there might have been diagnostic interaction. Highly limited rural access to medication is an effective impediment to its overuse among those with migraine or TTH, reducing propensity to MOH and the probability of diagnosis of pMOH rather than either antecedent headache.

The estimated prevalence of migraine in Cameroon (17.9%) closely matches that in Ethiopia (17.7% from a similar study [10]). Although somewhat lower than in several countries also with similar studies [19–22], including Zambia (22.9%) [9], it is higher than the estimated global prevalence of 14–15% [2]. Each of these estimates took account of both definite and probable migraine. The estimated prevalence of TTH (both definite and probable) in Cameroon (44.4%) is higher than estimates in many similar studies elsewhere [19–22], including both Ethiopia (20.6% [10]) and Zambia (22.8% [9]), and substantially higher than the estimated global prevalence of 26.0% [3]. Along with the high prevalences of pMOH and other H15 + , these findings leave no doubt that headache disorders are a major threat to population health in Cameroon. The extent to which this manifest as lost health will become known from estimates of attributable burden, which are yet to be reported.

### Strengths and limitations

This study used established methodology, in a large sample representative of the country. There were quality-control measures in place. These were clear strengths. As in all such cross-sectional studies, there were the limitations of dependence on recall, with diagnoses based solely on responses to a diagnostic question set. Ideally, this question set should be directly validated in the population of interest and in the local translation. Where lack of resources (in particular, lack of headache specialists) preclude the necessary re-interview of a subsample of participants, the question set needs to have been found reliable elsewhere. The HARDSHIP question set had been used previously in 20 countries and almost as many languages, and directly validated in four [23–26].

We did not consider malaria, a prominent cause of secondary headache. As noted earlier, if it occurred as a cause of headache among our participants, this would probably have been categorized as other H15 + . Data collection was completed prior to the SARS-CoV-2 (COVID-19) pandemic, so this was not a factor.

## Conclusion

This first population-based study of its prevalence in Cameroon found headache to be common. The estimate for migraine was broadly in keeping with those from other countries in SSA, while higher than the global mean. Headache on ≥ 15 days/month was very highly prevalent. These are matters for health policy, since these are painful disorders, and the findings are clearly indicative of unmet treatment need. But, in a country with limited resources and many calls upon them, measures of attributed burden (to be reported later) are needed to establish priority for resource allocation.

## Data Availability

The original data are held at Clinical Research Education, Networking and Consultancy (CRENC), Yaoundé, Cameroon, and the analytical set at Norwegian University of Science and Technology, Trondheim, Norway. When analyses are completed, anonymised data will be available on request for academic purposes, in line with the policy of the Global Campaign against Headache.

## Abbreviations

aOR: Adjusted odds ratio
CI: Confidence interval
CRENC: Clinical Research Education, Networking and Consultancy
d/m: Days/month
GBD: Global Burden of Disease
HARDSHIP: Headache-Attributed Restriction, Disability, Social Handicap and Impaired Participation questionnaire
HY: Headache yesterday
ICHD: International Classification of Headache Disorders
LTB: *Lifting* *The* *Burden*
MOH: Medication-overuse headache
OR: Odds ratio
OTC: Over-the-counter
pMOH: Probable MOH
SD: Standard deviation
SSA: Sub-Saharan Africa
TTH: Tension-type headache
USD: United States dollar
WHO: World Health Organization
XAF: Central African franc
